# Bending Elasticity Modulus of Giant Vesicles Composed of *Aeropyrum Pernix* K1 Archaeal Lipid

**DOI:** 10.3390/life5021101

**Published:** 2015-03-26

**Authors:** Julia Genova, Nataša Poklar Ulrih, Veronika Kralj-Iglič, Aleš Iglič, Isak Bivas

**Affiliations:** 1 Institute of Solid State Physics, Bulgarian Academy of Sciences, 72, Tzarigradsko Chaussee Blvd., 1784 Sofia, Bulgaria; E-Mails: ulia@issp.bas.bg (J.G.); bivas@issp.bas.bg (I.B.); 2 Biotechnical Faculty, University of Ljubljana, Jamnikarjeva 101, 1000 Ljubljana, Slovenia; E-Mail: natasa.poklar@bf.uni-lj.si; 3 Centre of Excellence for Integrated Approaches in Chemistry and Biology of Proteins (CipKeBiP), Jamova 39, SI-1000 Ljubljana, Slovenia; 4 Laboratory of Clinical Biophysics, Faculty of Health Sciences, University of Ljubljana, Zdravstvena 5, SI-1000 Ljubljana, Slovenia; 5 Laboratory of Biophysics, Faculty of Electrical Engineering, University of Ljubljana, Tržaška 25, SI-1000 Ljubljana, Slovenia; E-Mail: ales.iglic@fe.uni-lj.si

**Keywords:** giant vesicles, *Aeropyrum pernix* K1, archaeal lipids, membrane bending elasticity, membrane bending elasticity modulus, phospholipid bilayer, flickering, cell shapes

## Abstract

Thermally induced shape fluctuations were used to study elastic properties of giant vesicles composed of archaeal lipids C_25,25_-archetidyl (glucosyl) inositol and C_25,25_-archetidylinositol isolated from lyophilised *Aeropyrum pernix* K1 cells. Giant vesicles were created by electroformation in pure water environment. Stroboscopic illumination using a xenon flash lamp was implemented to remove the blur effect due to the finite integration time of the camera and to obtain an instant picture of the fluctuating vesicle shape. The mean weighted value of the bending elasticity modulus *k*_c_ of the archaeal membrane determined from the measurements meeting the entire set of qualification criteria was (1.89 ± 0.18) × 10^−19^ J, which is similar to the values obtained for a membrane composed of the eukaryotic phospholipids SOPC (1.88 ± 0.17) × 10^−19^ J and POPC (2.00 ± 0.21) × 10^−19^ J. We conclude that membranes composed of archaeal lipids isolated from *Aeropyrum pernix* K1 cells have similar elastic properties as membranes composed of eukaryotic lipids. This fact, together with the importance of the elastic properties for the normal circulation through blood system, provides further evidence in favor of expectations that archaeal lipids could be appropriate for the design of drug delivery systems.

## 1. Introduction

Archaeal diether lipids are recently attracting increased interest due to their potential role as drugs, genes, or cancer imaging agents [1]. In comparison with eukaryotic phospholipids, archaeal diether phospholipids contain branched fully saturated chains which are linked to glycerol with ether bonds [2]. These structural characteristics render archaeal phospholipids and their aggregates resistant to high temperatures, high concentrations of ions in solution and degradation by eukaryotic enzymes. It is therefore indicated that they would be persistent enough to deliver encapsulated substances to their target before being decomposed in body fluids or captured by the cells of the immune system. Furthermore, the carrier vehicles should be able to bring the contents into the target cells, which means that they have to interact with the host membranes. In order to design useful vehicles surrounded by membranes composed of archaeal lipids, it is necessary to study the properties of archaeal lipid membranes. Giant vesicles composed of lipid molecules are a convenient system to study the membrane properties as they can be prepared from natural or synthetic lipids using various formation techniques [3,4,5,6] and are large enough to be observed under the optical microscope. Knowing the elastic properties of lipid membranes in water environment one can elaborate the system of research and study the influence of different biologically relevant admixtures, such as proteins [6,7], hydrocarbons [8], acids [9], *etc.*, and combination of them, to get closer to real biological systems.

**Figure 1:**
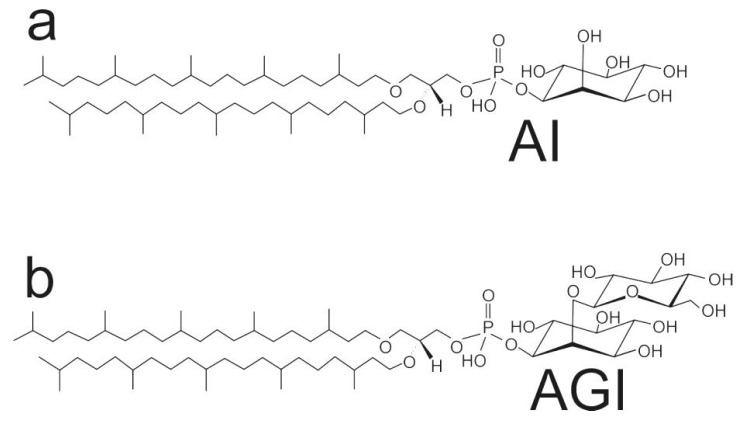
(**a**) Structural formulas of 2,3-di-*O*-sesterpanyl-*sn*-glycerol-1-phospho-myo-inositol (C_25,25_-archetidylinositol) (top: **AI**); (**b**) 2,3-di-*O*-sesterpanyl-*sn*-glycerol-1-phospho-1'-(2'*O*-α-d-glucosyl)-myo-inositol (C_25,25_-archetidyl (glucosyl) inositol) (bottom: **AGI**).

Recently, we characterized liposomes prepared from polar lipids isolated from *Aeropyrum pernix* K1 physicochemically [10,11]. Lipids of *Aeropyrum pernix* K1 are different from those of the anaerobic sulfur-dependent hyperthermophilic archaea due to a lack of both tetraether lipids and direct linkages of inositol and sugar moieties [12]. The isolated polar lipids of *A. pernix* consist solely of 2,3-di-*O*-sesterterpanyl-*sn*-glycerol (C_25,25_-archaeol). Their two major polar lipids are 2,3-di-*O*-sesterterpanyl-*sn*-glycerol-1-phospho-1'-(2'-*O*-α-D-glucosyl)-*myo*-inositol (C_25,25_-archaetidyl (glucosyl) inositol; AGI; about 91 mol %) and 2,3-di-*O*-sesterterpanyl-*sn*-glycerol-1-phospho-*myo*-inositol (C_25,25_-archaetidylinositol; AI; about 9 mol %) [12] (Figure 1).

Like eukaryotic lipids, archaeal lipids can form liposomes [13,14] and giant vesicles (GVs) [15,16]. The nonspecific properties of archaeal and eukaryotic lipids are similar enough for GVs to be formed from mixtures of archaeal and eukaryotic phospholipids [16].

The physicochemical properties of archaeal lipids are important in design of vaccine adjuvants [17,18] and drug carriers [13]. The design of convenient aggregates requires an understanding of the interactions between constituents on the microscopic level while in description of the membrane as a thin elastic shell, its properties are characterized by elastic moduli. The membrane of giant phospholipid vesicle poorly resists stretching, compression and rupture when exposed to such deformation. Subjected to external forces, the membrane rather undergoes a bending deformation. The energy required to deform the membrane is expressed by the membrane bending modulus *k*_c_. Statistical mechanical derivation of the phospholipid bilayer membrane free energy [19,20] links thermodynamic bending elasticity modulus of the membrane *k*_c_ [21] to the microscopic properties of the constituents. Local membrane bending affects lateral distribution of membrane proteins, their conformation, and consequently their functional role such as permeability for different substances and ability to bind particular ligands. Thereby vital processes of cell membrane can be connected to its bending modulus. The bending elasticity modulus is a material constant which does not depend on the size and shape of the object, but on the composition of the vesicle membrane and aqueous solution used. Recent studies on the relation between cell membrane elastic properties, and cell functions within a variety of biological cells shows a tight correlation between the cell membrane elasticity and the functions of these cells. A change in the bending elasticity modulus between resting and activated forms is found for different cell types, which is another evidence for the role of the elastic properties for the proper functioning of biological cell [22].

In eukaryotic GVs, shape fluctuations have been used to determine *k*_c_ [23,24,25,26,27], while to the best of our knowledge, no such measurement has hitherto been reported in archaeal GVs. In this work we determined the bending elasticity modulus of the archaeal bilayer membrane by measurement and mathematical analysis of the thermal shape fluctuations of archaeal GVs.

## 2. Materials and Methods

### 2.1. Bending Elasticity of Lipid Membrane

We shall assume that the ground state of the membrane is flat and unstretched. One deformed (curved) membrane is fully described at every point of its surface by its principal curvatures *c*_1_ and *c*_2_.

Let us consider a small patch of the deformed membrane with principal curvatures *c*_1_ and *c*_2_. It’s bending elastic energy per unit area *F*_c_ can be expressed according to Helfrich [28] via the relation:

(1) $$F_{c}=\frac{1}{2}k_{c}{\left(c_{1}+c_{2}-c_{0}\right)}^{2}+{\bar{k}}_{c}c_{1}c_{2}$$

where, *c*_0_ is the spontaneous curvature, $k_{c}$ is the bending elasticity modulus and ${\bar{k}}_{c}$ is the saddle bending elasticity modulus of the lipid bilayer. The spontaneous curvature of a symmetric membrane in a symmetric environment vanishes, *c*_0_ = 0.

### 2.2. Thermally Induced Shape Fluctuation Method

After the first detailed theoretical model of thermally induced shape fluctuations was proposed by Milner and Safran [29], experimental procedures based on the analysis of thermally induced shape fluctuations of quasi-spherical vesicles were developed for precise measurements of the bending elastic modulus [23,30]. The fundamental expression used by the authors is [23]:

(2) $$\langle {\left|U_{n}^{m}(t)\right|}^{2}\rangle =\frac{k_{B}T}{k_{c}}\frac{1}{\left(n-1)(n+2)[\bar{\sigma }+n(n+1)\right]}$$

where $\langle {\left|U_{n}^{m}(t)\right|}^{2}\rangle$ is the mean squared amplitude of the membrane fluctuations’ decomposition in spherical harmonics *Y*_n_^m^(*θ,φ*), *k*_B_ is the Boltzmann’s constant, *T* is the absolute temperature, *m* and *n* are the numbers, characterizing the given mode and $\bar{\sigma }=\sigma R^{2}/k_{c}$ is the dimensionless membrane tension (an adjustable parameter depending on the membrane tension and the difference of the lipid molecules in the inner and the outer layer of the lipid bilayer).

In fact, what is measured in an experiment of fluctuating quasi-spherical giant vesicle is the equatorial cross section radius in 128 or 64 (depending on the vesicle radius) equidistant directions from the center of the vesicle for every recorded image. In spherical coordinates the radius of the vesicle in the given direction can be written by the expression:

(3) $$\rho (\phi ,t)=R[1+u(\frac{\pi }{2},\phi ,t)]$$

where *R* represents the radius of a sphere with equal volume and u(θ=π/2,φ,t) is a function describing the membrane fluctuations, θ=π/2 represents the equatorial cross section of the vesicle with the plane, passing through the vesicle center, parallel to *xy* plane of the coordinate system. It was assumed that the amplitudes of the fluctuations are small compared to the vesicle radius, |u(θ,φ,t)|≪1. The normalized angular autocorrelation function of the vesicle radius is given by the expression:

(4) $$\xi (\gamma ,t)=\frac{1}{R^{2}}[\frac{1}{2\pi }\int_{0}^{2\pi }\rho (\phi +\gamma ,t)\rho *(\phi ,t)d\phi -\rho^{2}(t)]$$

It is shown in [23] that the time averaged angular autocorrelation function can be decomposed into Legendre polynomials with amplitudes *B_n_*, related to the mean squared amplitudes of spherical harmonics:

(5) $$B_{n}=\frac{2n+1}{4\pi }\langle {\left|U_{n}^{m}(t)\right|}^{2}\rangle$$

Taking into account the relations (4), (5) and (2) we can calculate the bending elasticity modulus of the membrane from the decomposition of the angular autocorrelation function of the equatorial cross-section radius as following:

(6) $$k_{c}=\frac{1}{B_{n}}\frac{k_{B}T}{4\pi }\frac{\left(2n+1\right)}{\left(n-1)(n+2)[\bar{\sigma }+n(n+1)\right]}$$

In all the experimental data obtained in this work stroboscopic illumination was used to remove the artifact due to the integration time of the video camera. The stroboscopically illuminated sample presents an instant picture of the object to the observer [31,32].

An algorithm for digitalization and processing of image sequences of fluctuating vesicles with a detailed procedure for obtaining the mechanical constants of the vesicular membrane, applying strict objective criteria for qualification of the vesicle as a whole as well as for acceptance or rejection of a given contour of the sequence of recorded images [32] was used for all the experimental data presented in the work. The white noise contribution to the amplitudes of thermal shape fluctuations [32] was evaluated and taken into account in the reported values for the bending elasticity modulus.

### 2.3. Experimental Section

#### 2.3.1. Chemicals

SOPC (1-stearoyl-2-oleoyl-sn-glycero-3-phosphocholine) and POPC (1-palmitoyl-2-oleoyl-sn-glycero-3-phosphocholine) were purchased from Avanti Polar Lipids, Inc. (Alabaster, AL, USA).

#### 2.3.2. Growth of *Aeropyrum Pernix* K1

*Aeropyrum pernix* K1 was purchased from the Japan Collection of Microorganisms (N° 9820; Wako-shi, Japan). The culture medium comprised (per liter): 34.0 g marine broth 2216 (DifcoTM Becton, Dickinson and Co., Franklin Lakes, NJ, USA), 5.0 g Trypticase Pepton (Becton, Dickinson and Company, Sparks, NV, USA), 1.0 g yeast extract (Becton, Dickinson and Company, Sparks, NV, USA) and 1.0 g Na_2_S_2_O_3_·5H_2_O (Sigma-Aldrich, St. Louis, MO, USA). The buffer systems used were 20 mM MES [2-(N-morpholino)ethanesulfonic acid; Acros Organics, Geel, Belgium] for growth at pH 6.0, and 20 mM HEPES [4-(2-hydroxyethyl)-1-piperazineethanesulfonic acid; Sigma-Aldrich Chemie GmbH, Steinheim, Germany] for growth at pH 7.0 and pH 8.0. The *A. pernix* cells were grown in 800 mL growth medium in 1000 mL thick-walled flasks, with a magnetic stirring hot plate and forced aeration (0.5 L·min^−1^) at 92 °C, as described previously [33].

#### 2.3.3. Isolation and Purification of Lipids

The polar lipid methanol fraction (PLMF) that is composed of approximately 91% 2,3-di-*O*-sesterpanyl-*sn*-glycerol-1-phospho-1'-(2'*O*-α-D-glucosyl)-myo-inositol (C_25,25_-archetidyl (glucosyl) inositol—**AGI**) and 9% 2,3-di-*O*-sesterpanyl-*sn*-glycerol-1-phospho-myo-inositol (C_25,25_-archetidylinositol—**AI**) with average molecular weight of 1181.42 g/moL was prepared from lyophilised *A. pernix* cells as described previously [10].

#### 2.3.4. Formaton of Giant Vesicles

**Figure 2:**
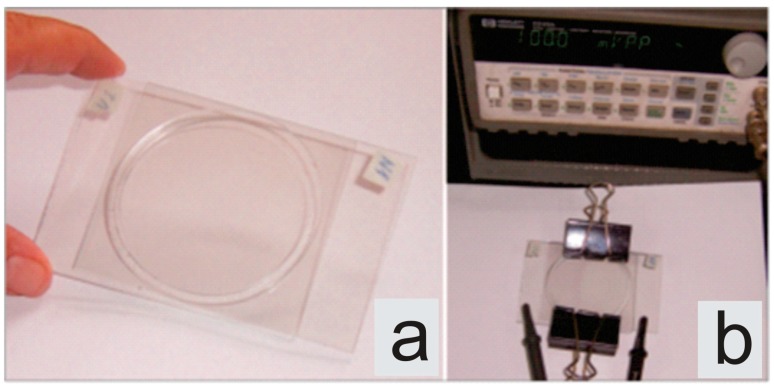
An image of the electroformation cell: (**a**) disconnected, (**b**) connected to the generator.

The giant vesicles were prepared using a modified electroformation method [3]. The electroformation cell used for all experimental procedures is shown in Figure 2. Glass slides, coated with a transparent conductor of indium tin oxide (ITO; thickness 100 *±* 20 nm, resistivity of 100 Ω*/*square) acted as electrodes. The lipid was dissolved in chloroform in concentration 1mg/mL. A number of small drops of the lipid solution were placed on the surface of the glass of the experimental cell in order to obtain as much lipid deposit for vesicle formation as possible. The prepared glass slides were put into an evacuated chamber for about 30 minutes. After evaporation of the solvent the experimental cell was filled with fresh double distilled (via quartz distiller) and filtered through Minisart 16534 syringe filter (pore size 0.20 µm) water. A low frequency sinusoidal alternating voltage (10 Hz, 1.5 V peak to peak) was applied to the conductive glass slides overnight. This procedure leads to the formation of vesicles appropriate for our experiment—*i.e.*, fluctuating vesicles with a diameter of the order of 20–40 µm with no visible defects.

#### 2.3.5. Observation and Recording of Giant Vesicles

**Figure 3:**
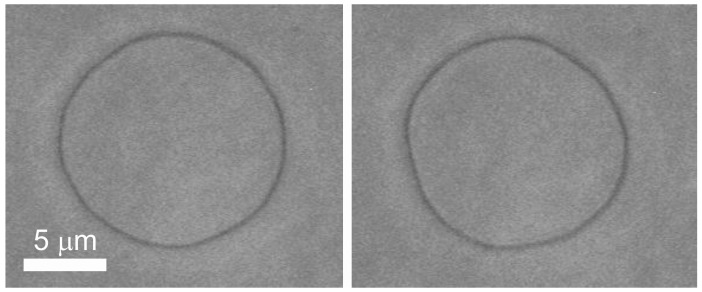
Images of the equatorial cross-section of a fluctuating vesicle at two times as observed by the phase contrast microscope.

Samples of the fluctuating giant vesicles were observed under a phase contrast microscope (Axiovert 100, Zeiss, Germany, oil immersion objective Ph3 100× magnification). Stroboscopic illumination comprising of an L6604 xenon flash lamp, an E7289-01 external main discharge capacitor and a C6096 power supply, all from Hamamatsu, Japan) [31] and a damping vibration system was used in the experiment [32]. The flash of the stroboscopic illumination was synchronized with the vertical pulses coming from the CCD video camera controller (C2400-60, Hamamatsu, Japan). According to the Hamamatsu data sheet, the light pulses were of less than 3–4 μs duration (full width at half maximum) at 2 J input energy. A double channel thermostatic stage was used to ensure that the bending elasticity measurements were done at a constant temperature (27 ± 0.1) °C. Fluctuating quasispherical (the deviations from spherical shape are small in comparison with the mean sphere radius) vesicles without visible defects with diameters 20–40 µm were chosen for the experiment. For each vesicle, approximately 400 images of its equatorial cross section (see Figure 3) were recorded with a frequency of 1 image per second and analyzed for determining the membrane bending elasticity moduli and tensions [34].

## 3. Results and Discussion

The analysis of thermally induced shape fluctuations of giant vesicles was used to determine the bending elasticity modulus of giant vesicles composed of archaeal lipids.

The experimental data obtained for the bending elasticity modulus *k*_c_ for pure archaeal lipid membranes in a water environment is presented in Table 1. For comparison, the corresponding values for synthetic POPC and SOPC membranes are given, measured by the same method in the same environment (pure water). The value of the bending elasticity modulus of the archaeal lipid membrane was calculated as the weighted average value of an ensemble of giant vesicles, meeting all the imposed qualification criteria. Our results indicate that lipids isolated from archaea *Aeropyrum pernix* K1 have similar bending elasticity modulus as eukaryotic lipid POPC and SOPC membranes. To the best of our knowledge, data on the measured modulus of bending elasticity of the archaeal membrane is here reported for the first time. The bending elasticity modulus of the membrane depends on the temperature below and near the phase transition temperature, but far above the phase transition temperature (this is the case in our experiment) the bending elasticity modulus of the membrane is practically constant [35].

**Table 1:** Bending elasticity modulus of isotropic membranes *k*_c_ composed of *Aeropyrum pernix* K1 archaeal lipid and eukaryotic lipids 1-stearoyl-2-oleoyl-sn-glycero-3-phosphocholine (SOPC) and 1-palmitoyl-2-oleoyl-sn-glycero-3-phosphocholine (POPC).

Type of Lipid	Weighted Mean Value of the Bending Elasticity Modulus *k*_c_ (± Standard Deviation)
*Aeropyrum pernix* K1 archaeal lipid	(1.89 ± 0.18) × 10^−19^ J
SOPC	(1.88 ± 0.17) × 10^−19^ J
POPC	(2.00 ± 0.21) × 10^−19^ J

The role of archaeal lipids as vaccine adjuvants and the possibility of using liposomes formed from archaeal lipids as delivery systems for drugs, genes and proteins provide incentives to investigate the interactions between archaeal and eukaryotic lipids. Furthermore, our previous studies indicated that liposomes can be formed from mixtures of standard and archaeal lipids [16] which would enable the creation of new delivery systems with lower contents of archaeal lipids.

The bending elasticity, which resides in the elastic deformability of red blood cells as well as drug delivery capsules, has substantial influence on the arterioles and the capillaries blood flow and has an important impact on normal circulation through blood system. These facts provide further evidence in favor of expectations that archaeal lipids could be appropriate for the design of drug delivery systems [36].

## 4. Conclusions

Comparing the results obtained for the bending elasticity modulus of the archaeal lipid membrane with that of a pure synthetic POPC and SOPC lipid membranes (all in a pure water environment), we can conclude that all three types of membranes have similar properties with respect to bending elasticity. From our study it follows that, at room temperature, the thermophilicity is not necessarily related to the bending elasticity.

## References

[1] Moghimipour E., Kargar M., Ramezani Z., Handali S. (2013). The potent *in vitro* skin permeation of archaeosome made from lipids extracted of sulfolobus acidocaldarius. Archaea.

[2] Corcelli A., Lobasso S. (2006). Characterization of lipids of halophilic archaea. Methods Microbiol..

[3] Angelova M.I., Soléau S., Méléard P., Faucon F., Bothorel P., Helm C., Lösche M., Möhwald H. (1992). Preparation of giant vesicles by external AC electric fields. Kinetics and applications. Trends in Colloid and Interface Science VI.

[4] Bagatolli L.A., Parasassi T., Gratton E. (2000). Giant phospholipid vesicles: Comparison among the whole lipid sample characteristics using different preparation methods: A two photon fluorescence microscopy study. Chem. Phys. Lipids.

[5] Peterlin P., Arrigler V. (2008). Electroformation in a flow chamber with solution exchange as a means of preparation of flaccid giant vesicles. Colloids Surf. B Biointerfaces.

[6] Pavlič J.I., Genova J., Zheliaskova A., Iglič A., Mitov M.D. (2010). Electroformation of neutral and negatively charged phospholipid giant vesicles under physiological conditions. C. R. L’Academie Bulg. Sci..

[7] Vitkova V., Mader M.A., Polack B., Misbach C., Podgorski T. (2006). Micro-macro link in rheology of erythrocyte and vesicle suspensions. Biophys. J..

[8] Genova J., Zheliaskova A., Mitov M.D. (2007). Monosaccharides (fructose, glucose) and disaccharides (sucrose, trehalose) influence the elasticity of SOPC membranes. J. Optoelec. Adv. Mater..

[9] Mitkova D., Marukovich N., Ermakov Y.A., Vitkova V. (2014). Bending rigidity of phosphatidylserine-containing lipid bilayers in acidic aqueous solutions. Colloids Surf. A Physicochem. Eng. Asp..

[10] Ulrih N.P., Gmajner D., Raspor P. (2009). Structural and physicochemical properties of polar lipids from thermophilic archaea. Appl. Microbiol. Biotechnol..

[11] Gmajner D., Ota A., Sentjurc M., Ulrih N.P. (2011). Stability of diether C-25,C-25 liposomes from the hyperthermophilic archaeon *Aeropyrum pernix* K1. Chem. Phys. Lipids.

[12] Morii H., Yagi H., Akutsu H., Nomura N., Sako Y., Koga Y. (1999). A novel phosphoglycolipid archaetidyl(glucosyl)inositol with two sesterterpanyl chains from the aerobic hyperthermophilic archaeon *Aeropyrum pernix* K1. Biochim. Biophys. Acta.

[13] Sprott G.D., Tolson D.L., Patel G.B. (1997). Archaeosomes as novel antigen delivery systems. FEMS Microbiol. Lett..

[14] Patel G.B., Sprott G.D. (1999). Archaeobacterial ether lipid liposomes (archaeosomes) as novel vaccine and drug delivery systems. Crit. Rev. Biotechnol..

[15] Bagatolli L., Gratton E., Khan T.K., Chong P.L.G. (2000). Two-photon fluorescence microscopy studies of bipolar tetraether giant liposomes from thermoacidophilic archaebacteria Sulfolobus acidocaldarius. Biophys. J..

[16] Sustar V., Zelko J., Lopalco P., Lobasso S., Ota A., Ulrih N.P., Corcelli A., Kralj-iglic V. (2012). Morphology, biophysical properties and protein-mediated fusion of archaeosomes. PLoS One.

[17] Sprott G.D., Dicaire C.J., Cote J.-P., Whitfield D.M. (2008). Adjuvant potential of archaeal synthetic glycolipid mimetics critically depends on the glyco head group structure. Glycobiology.

[18] Kamath A.T., Rochat A.-F., Christensen D., Agger E.M., Andersen P., Lambert P.-H., Siegrist C.-A. (2009). A Liposome-based mycobacterial vaccine induces potent adult and neonatal multifunctional T cells through the exquisite targeting of dendritic cells. PLoS One.

[19] Bivas I., Hanusse P., Bothorel P., Lalanne J., Aguerrechariol O. (1987). An application of the optical microscopy to the determination of the curvature elastic-modulus of biological and model membranes. J. Phys..

[20] Bivas I., Bivolarski L., Mitov M., Derzhanski A. (1992). Correlations between the form fluctuation modes of flaccid quasispherical lipid vesicles and their role in the calculation of the curvature elastic modulus of the vesicle membrane. Numerical results. J. Phys. II.

[21] Bivas I., Meleard P. (2003). Bending elasticity and bending fluctuations of lipid bilayer containing an additive. Phys. Rev. E.

[22] Pontes B., Ayala Y., Fonseca A.C.C., Romao L.F., Amaral R.F., Salgado L.T., Lima F.R., Farina M., Viana N.B., Moura-Neto V. (2013). Membrane elastic properties and cell function. PLoS One.

[23] Bivas I. (2010). Shape fluctuations of nearly spherical lipid vesicles and emulsion droplets. Phys. Rev. E.

[24] Faucon J., Mitov M., Meleard P., Bivas I., Bothorel P. (1989). Bending elasticity and thermal fluctuations of lipid-membranes—Theoretical and experimental requirements. J. Phys..

[25] Meleard P., Gerbeaud C., Pott T., Fernandez-Puente L., Bivas I., Mitov M.D., Dufourcq J., Bothorel P. (1997). Bending elasticities of model membranes: Influences of temperature and sterol content. Biophys. J..

[26] Vitkova V., Genova J., Meleard P. (2003). Influence of alamethicin on the passive water permeability of model lipid membranes and on the morphology of giant lipid vesicles. J. Mater. Sci..

[27] Genova J., Zheliaskova A., Vitkova V., Mitov M.D. (2009). Stroboscopic illumination study of the dynamics of fluctuating vesicles. J. Optoelectron. Adv. Mater..

[28] Helfrich W. (1973). Elastic properties of lipid bilayers—Theory and possible experiments. Z. Naturforschung C.

[29] Milner S., Safran S. (1987). Dynamic fluctuations of droplet microemulsions and vesicles. Phys. Rev. A.

[30] Mitov M.D., Faucon J.F., Meleard P., Bivas I., Bothorel P., Gokel G.W. (1992). Thermal fluctuations of membranes. Advances in Supramolecular Chemistry.

[31] Genova J., Vitkova V., Aladjem L., Meleard P., Mitov M. (2005). Stroboscopic illumination gives new opportunities and improves the precision of bending elastic modulus measurements. J. Optoelectron. Adv. Mater..

[32] Genova J., Pavlič J.I. (2012). Realization of Marin Mitov idea for the stroboscopic illumination used in optical microscopy. Bulg. J. Phys..

[33] Milek I., Cigić B., Skrt M., Kaletunç G., Ulrih N.P. (2005). Optimization of growth for the hyperthermophilic archaeon *Aeropyrum pernix* on a small-batch scale. Can. J. Microbiol..

[34] Genova J., Vitkova V., Bivas I. (2013). Registration and analysis of the shape fluctuations of nearly spherical lipid vesicles. Phys. Rev. E.

[35] Fernandez-Puente L., Bivas I., Mitov M.D., Meleard P. (1994). Temperature and chain length effects on bending elasticity of phosphatidylcholine bilayers. Europhys. Lett..

[36] Bedina Zavec A., Ota A., Zupančič T., Komel R., Poklar Ulrih N., Liović M. (2014). Archeosomes can efficiently deliver different types of cargo into epithelial cells grown *in vitro*. J. Biotechnol..

